# Tau acts as an independent genetic risk factor in pathologically proven PD

**DOI:** 10.1016/j.neurobiolaging.2011.11.001

**Published:** 2012-04

**Authors:** Gavin Charlesworth, Sonia Gandhi, Jose M. Bras, Roger A. Barker, David J. Burn, Patrick F. Chinnery, Stephen M. Gentleman, Rita Guerreiro, John Hardy, Janice L. Holton, Andrew Lees, Karen Morrison, Una-Marie Sheerin, Nigel Williams, Huw Morris, Tamas Revesz, Nicholas W. Wood

**Affiliations:** a Department of Molecular Neuroscience, UCL Institute of Neurology, Queen Square, London, UK; b Department of Clinical Neurosciences, Addenbrooke's Hospital, University of Cambridge, Cambridge, UK; c Institute for Ageing and Health, Newcastle University, Clinical Ageing Research Unit, Campus for Ageing and Vitality, Newcastle upon Tyne, UK; d Institute for Ageing and Health, Newcastle University, Mitochondrial Research Group, Newcastle upon Tyne, UK; e Neuropathology Unit, Centre for Neuroscience, Division of Experimental Medicine, Department of Medicine, Imperial College, London, UK; f Queen Square Brain Bank for Neurological Disorders, Institute of Neurology, University College London, London, UK; g Division of Neurosciences, University of Birmingham, Edgbaston, Birmingham, UK; h MRC Centre for Neuropsychiatric Genetics and Genomics, Cardiff University School of Medicine, Cardiff, UK; i UCL Genetics Institute, London, UK

**Keywords:** Genetics, Association study, Parkinson's disease, MAPT, Tau, Progressive supranuclear palsy, PD, PSP

## Abstract

*MAPT* has been repeatedly linked with Parkinson's disease (PD) in association studies. Although tau deposition may be seen in PD, its relevance to the pathogenesis of the condition remains unclear. The presence of tau-positive inclusions is, however, the defining feature of progressive supranuclear palsy (PSP), which may often be clinically misdiagnosed as idiopathic PD. On a genetic level, variants in *MAPT* are the strongest risk factor for PSP. These facts raise the question whether the *MAPT* association in PD results from contamination with unrecognized cases of PSP. Using only neuropathologically proven PD, we show that the *MAPT* association remains and is independent of the PSP Association.

## 1 Introduction

To date, several large-scale genome-wide association studies (GWAS) have been completed for Parkinson's disease (PD), culminating in a recently published meta-analysis that identified 16 loci surpassing the threshold for genome-wide significance (International Parkinson's Disease Genomics Consortium et al., 2011; International Parkinson's Disease Genomics Consortium and Wellcome Trust, Case Control Consortium 2, 2011; Satake et al., 2009; Simón-Sánchez et al., 2009; UK Parkinson's Disease Consortium et al., 2011). Of these 16 loci, some are predictable findings, while others offer the new insights into the pathogenesis of the disease. For example, from a neuropathological perspective, the discovery that variants in *SNCA* act as a risk factor for PD is consistent with the deposition of aggregated α-synuclein in the PD brain. The detection of variation in *MAPT* as a risk factor in PD was unexpected, as the relevance of tau aggregation in PD remains unclear.

Predictably, a number of tauopathies are associated with common or rare variants in *MAPT*, including corticobasal degeneration, frontotemporal dementia with parkinsonism linked to chromosome 17, and progressive supranuclear palsy (PSP) (Vandrovcova et al., 2009). In fact, variation in *MAPT* is the strongest genetic risk factor for PSP. The association with this condition is driven by a series of polymorphisms in near complete linkage disequilibrium, which form an extended haplotype, termed H1, that covers the entire gene (Baker et al., 1999). Inheritance of 2 copies of the risk haplotype in this region confers an odds ratio for developing PSP of approximately 4 (Baker et al., 1999).

There can be considerable clinical overlap between PD and PSP, especially in the earlier stages of the diseases. Even with increased awareness, the rate of misdiagnosis of idiopathic PD has been estimated at between 10% and 25% in autopsy studies, and 6%–26% in community studies (Hughes et al., 1992; Newman et al., 2009). In one autopsy study of 100 cases of clinically diagnosed PD, 24 cases were found to be misdiagnosed and 6 of these were PSP (Hughes et al., 1992). It is probable that large GWAS, using clinically diagnosed PD cases, will contain some individuals with PSP. Despite the potential for clinical confusion, PSP is neuropathologically distinct from idiopathic PD. PSP is classified as a primary tauopathy and is characterized morphologically by deposition of 4-repeat tau in neurons as neurofibrillary tangles and in both astrocytes and oligodendroglia as tufted astrocytes and coiled bodies, respectively. The morphological characteristics and anatomical distribution of the tau pathology and the biochemical composition of the tau lesions are different from those seen in Alzheimer's disease (Hauw et al., 1994). PD, on the other hand, is classified as a synucleinopathy, characterized by abnormal fibrillar cytoplasmic inclusions, termed Lewy bodies, of which the principal protein component is α-synuclein (Braak et al., 2003).

Given the strength of the association between *MAPT* with PSP, it has been suggested that the tau signal seen in Parkinson's disease association studies might be the result of unrecognized PSP contamination of the case cohort. Therefore, in order to answer this important question, we performed a focused analysis of our pathologically proven PD cases from our recent GWAS datasets (International Parkinson's Disease Genomics Consortium et al., 2011).

## 2 Methods

Cases were selected from two brain banks in London with a confirmed primary neuropathological diagnosis of PD and genotype data from the discovery or replication phase of our recent meta-analysis. Neuropathological diagnosis had been made by an experienced neuropathologist and was based on accepted morphological criteria (Ince et al., 2008). Two hundred forty-five pathologically proven PD cases and 5445 controls from the discovery phase and 140 cases and 4537 controls from the replication phase of our published meta-analysis were suitable for this analysis. Some of the pathological samples were used in previous studies (De Silva et al., 2002; Vandrovcova et al., 2009).

The primary analysis focused on a region approximately 500 kilobase (kb) either side of *MAPT*. The single nucleotide polymorphisms (SNPs) tested for association in this region were selected on the basis that they had achieved genome-wide significance in the UK samples used in the discovery phase of our GWAS meta-analysis and had also been genotyped on the custom-built immunochip used for the replication phase. Eight SNPs met these requirements (rs393152, rs1635291, rs7215239, rs1237319, rs17690703, rs17769552, rs1981997, and rs8070723). None of the SNPs in *MAPT* chosen by this method are in significant linkage disequilibrium with the SNPs used by Vandrocova et al. (2009) in their *MAPT* haplotype analysis (maximum *r*^2^ = 0.2).

In earlier GWAS, the region surrounding α-synuclein (*SNCA*) has consistently been shown to be the locus most strongly associated with PD (International Parkinson's Disease Genomics Consortium et al., 2011; UK Parkinson's Disease Consortium et al., 2011; Satake et al., 2009). Thus, in order to verify that this analysis had sufficient power to detect previously well-documented associations, we also included the SNPs in a second 1 megabase (Mb) region spanning *SNCA* that met the same requirements as above. Two SNPs were available for testing (rs356220 and rs2736990).

The association analyses for the discovery and replication datasets were performed separately using PLINK (Purcell et al., 2007). Subsequently, a meta-analysis of the 2 datasets was performed using the same software. Fixed and random effects models were used to generate *p*-values and the I^2^ index was used to quantify heterogeneity between the studies.

## 3 Results

**Fig. 1:**
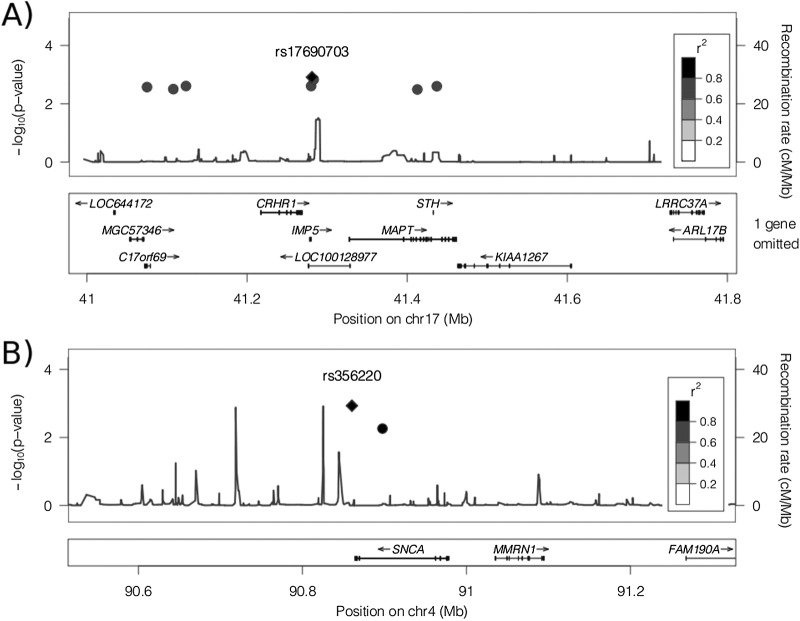
Graphical representation of the physical position of SNPs tested across the MAPT (A) and SNCA (B) loci, with *p*-values and linkage disequilibrium (LD). A diamond represents the top hit, which is the reference for the LD calculations. Recombination rate in centiMorgan per megabase (cMMb) is plotted against the right-hand axis.

After review of the reports, 385 cases of neuropathologically proven idiopathic PD remained for analysis. Two hundred forty-five were male and 140 female. The primary analysis demonstrated statistically significant association in the 8 SNPs spanning the *MAPT* gene (Table 1 and Fig. 1A). The SNP showing strongest association in this study was rs17690703, which had a *p*-value of 0.001225 and was calculated to confer an odds ratio of 0.75 per minor allele dose. Association at rs8070723 confirms previous data suggesting that the additional risk in PD is conferred by the H1 haplotype of the *MAPT* inversion (Simón-Sánchez et al., 2009). Further dissection of subhaplotypes was not possible with the limited number of SNPs included in this study. In particular, no proxy in sufficient linkage disequilibrium (*r*^2^ > 0.8) with the H1-specific SNP rs242557, which has previously been implicated in PSP but not PD, was available for testing (Wider et al., 2010).

The results of the secondary analysis using SNPs spanning *SNCA* confirms this study's ability to detect the predicted association with this region using only the relatively small subpopulation of neuropathologically confirmed cases of PD (Table 2 and Fig. 1B). Rs356220, the SNP showing the strongest association in this study, had a *p*-value of 0.001167 (fixed effects model) and was calculated to confer an odds ratio of 1.31 per minor allele dose. The I^2^ index of 0.35 indicated some heterogeneity between the 2 datasets, but was well below the standard cutoff point of 0.75. Furthermore, this SNP is in high linkage disequilibrium (*r*^2^ > 0.9) with and shows the same direction of effect as the top SNP from the largest meta-analysis published to date (rs356219; not included in this study as not common to both genotyping platforms) suggesting a consistent pattern of association (International Parkinson's Disease Genomics Consortium et al., 2011).

## 4 Discussion

Clinical misdiagnosis has the potential to introduce contamination into association analysis. With regard to PD GWAS, PSP contamination is of particular concern as the association between PSP and the *MAPT* locus is particularly strong. In this analysis, we relied solely on neuropathological diagnosis as the inclusion criterion for the case cohort.

The results confirm that the region surrounding *MAPT* is associated with idiopathic PD in our neuropathologically proven PD cohort and, therefore, that this association does not result from contamination with primary tauopathies, such as PSP. Although the gene most likely to be responsible for the association signal remains *MAPT* itself, this gene is located in a block of near complete linkage disequilibrium that extends over a total of nearly 2 Mb on chromosome 17. It is conceivable, therefore, that a different gene within this haplotype block may in fact be driving the association.

Accepting *MAPT* as the most likely candidate, the persistence of the association at this locus in pathologically proven PD raises the possibility that that dysfunction of tau may in fact be pathogenic in PD. Variants in *MAPT* may act either to increase expression or alter the splicing of tau so as to favor its aggregation. It is already known that the H1c haplotype, which underlies the risk in PSP, results in increased expression of tau, particularly of 4 repeat-containing transcripts (Myers et al., 2007). Recently, it has also become increasingly clear that, in many neurodegenerative diseases, the aggregation of one protein may often be associated with or even induce the aggregation of others (Higashi et al., 2007). On a genetic level, studies in clinically defined PD have suggested that genotypes at the *MAPT* and *SNCA* loci act synergistically to confer susceptibility to PD and that variation at *MAPT* may be particularly associated with cognitive decline (Goris et al., 2007; Williams-Gray et al., 2009). Certainly, Alzheimer-like tau pathology has long been known to coexist with the morphological changes typical of idiopathic PD. This has mostly been viewed as a consequence of pathological aging or coexistent early Alzheimer's disease, rather than as being an intrinsic part of the pathogenic pathway of PD itself. Coexistent Alzheimer's disease seems, however, unlikely to be able to fully explain the *MAPT* association in idiopathic PD as a recent large GWAS in Alzheimer's disease failed to demonstrate any association at this locus (Naj et al., 2011). Significant tau pathology has also been noted in some monogenic forms of PD, including in some cases resulting from *LRRK2* mutation and in some members of the Contursi kindred, who carry the A53T *SNCA* mutation, as well as in α-synuclein overexpressing transgenic mice (Devine and Lewis, 2008; Haggerty et al., 2011).

It is likely that more detailed neuropathological studies of tau pathology in the brains of individuals with idiopathic and genetic forms of PD will be required to fully dissect out its role in the pathogenesis of this condition. One promising approach may be to search for a correlation between the extent of tau pathology in idiopathic PD and the genotype at the *MAPT* locus. The existence of such a correlation would support the idea of a direct effect of these variants on tau aggregation.

## Disclosure statement

The authors have no conflicts of interest to declare.

All DNA samples were obtained in accordance with the approval of local ethics committees.

## Figures and Tables

**Table 1:** SNPs in a 1-Mb region spanning *MAPT* showing statistically significant association with *p* values in fixed and random effects models, I^2^ index, minor allele frequency (MAF) and odds ratio per minor allele dose

SNP reference	Physical position	*p* Value (fixed effects)	*p* Value (random effects)	I^2^ index	MAF	Odds ratio per minor allele dose
rs17690703	41281077	0.001225	0.001225	0.00	0.28	0.75
rs17769552	41283070	0.001485	0.001485	0.00	0.24	0.74
rs7215239	41123556	0.002481	0.002481	0.00	0.26	0.76
rs12373139	41279910	0.002484	0.002484	0.00	0.24	0.76
rs8070723	41436901	0.002529	0.002529	0.00	0.24	0.76
rs393152	41074926	0.002701	0.002701	0.00	0.24	0.76
rs1635291	41107696	0.003155	0.003155	0.00	0.26	0.77
rs1981997	41412603	0.003257	0.003257	0.00	0.24	0.76

Key: Mb, megabase; SNP, single nucleotide polymorphism.

**Table 2:** SNPs in a 1-Mb region spanning *SNCA* showing statistically significant association with *p* values in fixed and random effects models, I^2^ index, minor allele frequency (MAF), and odds ratio per minor allele dose

SNP reference	Physical position	*p* Value (fixed effects)	*p* Value (random effects)	I^2^ index	MAF	Odds ratio per minor allele dose
rs356220	90860363	0.001167	0.01414	0.35	0.36	1.28
rs2736990	90897564	0.005539	0.005539	0.00	0.45	1.23

Key: SNP, single nucleotide polymorphism.
